# Fully automated protein complex prediction based on topological similarity and community structure

**DOI:** 10.1186/1477-5956-11-S1-S9

**Published:** 2013-11-07

**Authors:** Chengwei Lei, Saleh Tamim, Alexander JR Bishop, Jianhua Ruan

**Affiliations:** 1 Department of Computer Science, The University of Texas at San Antonio, San Antonio, TX 78249, USA; 2 Greehey Children's Cancer Research Institute, The University of Texas Health Science Center at San Antonio, San Antonio, TX 78229, USA; 3 Department of Cellular and Structural Biology, The University of Texas Health Science Center at San Antonio, San Antonio, TX 78229, USA

**Keywords:** PPI network, random walk, protein-protein interaction, protein complex, clustering

## Abstract

To understand the function of protein complexes and their association with biological processes, a lot of studies have been done towards analyzing the protein-protein interaction (PPI) networks. However, the advancement in high-throughput technology has resulted in a humongous amount of data for analysis. Moreover, high level of noise, sparseness, and skewness in degree distribution of PPI networks limits the performance of many clustering algorithms and further analysis of their interactions.

In addressing and solving these problems we present a novel random walk based algorithm that converts the incomplete and binary PPI network into a protein-protein topological similarity matrix (PP-TS matrix). We believe that if two proteins share some high-order topological similarities they are likely to be interacting with each other. Using the obtained PP-TS matrix, we constructed and used weighted networks to further study and analyze the interaction among proteins. Specifically, we applied a fully automated community structure finding algorithm (Auto-HQcut) on the obtained weighted network to cluster protein complexes. We then analyzed the protein complexes for significance in biological processes. To help visualize and analyze these protein complexes we also developed an interface that displays the resulting complexes as well as the characteristics associated with each complex.

Applying our approach to a yeast protein-protein interaction network, we found that the predicted protein-protein interaction pairs with high topological similarities have more significant biological relevance than the original protein-protein interactions pairs. When we compared our PPI network reconstruction algorithm with other existing algorithms using gene ontology and gene co-expression, our algorithm produced the highest similarity scores. Also, our predicted protein complexes showed higher accuracy measure compared to the other protein complex predictions.

## Introduction

Protein-protein interaction (PPI) is the core to many fundamental biological processes. New high-throughput techniques, such as yeast two-hybrid and tandem affinity purification [1], have vastly increased the size of the protein-protein interaction data. With this large amount of protein-protein interaction (PPI) data which is usually modeled by PPI networks, the cell mechanistic can be understood at system level.

The growing PPI database helps both biological and computational scientists to predict gene functions, functional pathways, protein complexes and improve the diagnosis and treatment of diseases [2-16]. However, the growing of PPI network poses multiple challenges at the same time, such as PPI networks often have a high false positive rate and an even higher false negative rate [17]. PPI networks are also known to have skewed degree distribution, meaning that they have more than expected quantity of hub genes [18]. Additionally, the PPI networks are typically binary (sometimes with limited discrete value) and sparse, partially due to the high false negative rate, which places a hurdle for protein complex prediction.

In biological networks, community structures (protein complexes) normally have key biological significance. Identifying and analyzing such communities leads to the understanding of the nature of the system as well as potential novel relationships between communities [19]. One approach to identifying communities is using modularity-based method which aims at optimizing modularity (Q) and shown to be effective in measuring the overall quality of community structures [20]. Qcut [19] is an efficient heuristic algorithm that combines spectral graph partitioning and local search to optimize Q. It is able to find stronger community structures in large and relatively dense networks. For larger networks, the modularity function may face resolution limit problem since communities with relatively high intercommunity connectivity may be merged into a single community [21]. HQcut [19] solves this resolution limit problem by applying Qcut recursively to the communities that have already been identified.

Recently, we proposed a novel idea to predict protein-protein interactions based on topological similarity between nodes in a given PPI network [18,22]. Basically, we consider two nodes to be similar if they have similar distances to all other nodes in the network (instead of only their direct neighbors), measured by a novel random walk procedure. While the results suggest that the predicted edges likely represent true physical protein-protein interactions, the algorithm relies on an arbitrary similarity cutoff to distinguish between interacting vs non-interacting protein pairs. In particularly, given the sparsity of the known PPI networks, we believe the coverage of PPIs can be significantly improved if an optimal cutoff can be selected.

In this work, we extend our work to introduce a novel approach for determining an optimal cutoff to predict protein-protein interactions from the weighted topological similarity matrix. Our idea is that a good cutoff should result in a network that is highly modular. Based on this idea, we also present a parameter-free, recursive algorithm, Auto-HQcut, to identify protein complexes from the PPI network. We also developed a graphical user interface to enable visualization of the weighted network and the predicted protein complexes.

To evaluate our approach, we applied the network reconstruction algorithm to a yeast PPI network and examine the biological relevance of the resulting PPI network. The resulting PPI network showed interactions with more functional relevance in comparison to the original PPI network. Comparison with existing methods showed that the network reconstructed by our method has the highest overall quality. Furthermore, applying Auto-HQcut clustering algorithm, we found that the reconstructed network had significantly improved prediction accuracy of protein complexes.

## Results

For evaluation, we applied our algorithm to a yeast core PPI network obtained from [23], which covers 2708 genes with 7123 edges. By performing a modified random walk on this network and calculating similarities between every pair of nodes based on their topology equivalence, we derived a modified Protein-Protein Topological Similarity matrix (called PP-TS matrix), which covers all the topological similarities between any pair of genes in the network. To evaluate the functional relevance of the newly predicted pairwise similarities, we resort to two types of sources, gene ontology and gene expression.

For clustering, we used the methods based on the optimization of a modularity function (Q). We developed an approach, Auto-HQcut, to automatically determine the best parameter for the algorithm HQcut. We compared the clustering accuracy of each resulting protein complex to the known complexes. The results showed that our approach could be used to deduce the significant biological processes from any PPI network.

### Biological functional relevance

The performance of the algorithm can be tested by evaluating the pairwise genes with top similarity scores in the PP-TS matrix for functional relevance. It is expected for the gene pairs with high PP-TS scores to be functionally more relevant. To determine the functional relevance between the gene pairs, we used the functional annotations in gene ontology (GO) and gene expression (GE) patterns across diverse conditions.

**Figure 1:**
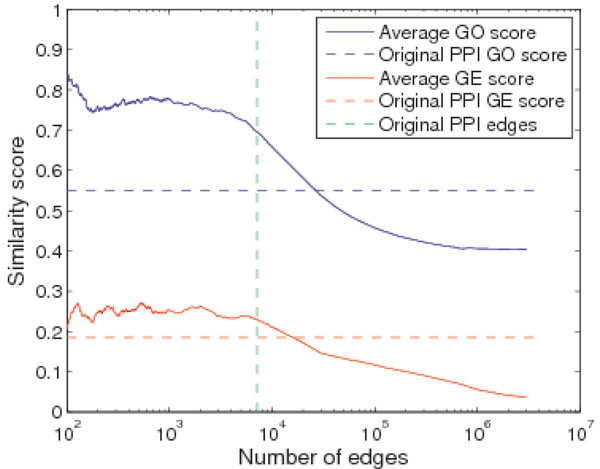
**Quality of the pairs of proteins with top PP-TS scores**. Predicted interactions are ranked by the PP-TS scores. All interactions above a particular rank are then used to calculate the average gene ontology similarity (GO) and gene co-expression (GE) score. Two horizontal reference lines are the average GO and GE score of all edges in the original PPI network. The vertical reference line represents the number of edges in the original PPI network.

To represent the functional relevance of the top gene pairs in the PP-TS matrix, we calculated the average gene ontology similarity (called Average GO score) and gene co-expression (called Average GE score) of the gene pairs with the top PP-TS scores, as shown in Figure 1.

For GO, we used the gene pairs with top PP-TS score to obtain the same average GO score as the original PPI network to identify more associated gene pairs (26170 compared to 7123) which were not available in the original network. On the other hand, if we keep the same number of edges ("Original PPI edges" in Figure 1) as in original PPI network, the average GO similarity score is improved from 0.5512 to 0.6956. Similar to the GO, for GE, keeping the same average GE score, we get more associated gene pairs (15480 compared to 7123). Keeping the same number of edges as in original PPI network, the average GE similarity score is improved from 0.1865 to 0.2295.

With these two independent sources of evidence, the results show that the gene pairs with higher topological similarity scores have higher biological functional relevance. The results also show a consistent pattern between gene ontology similarity and gene co-expression.

**Figure 2:**
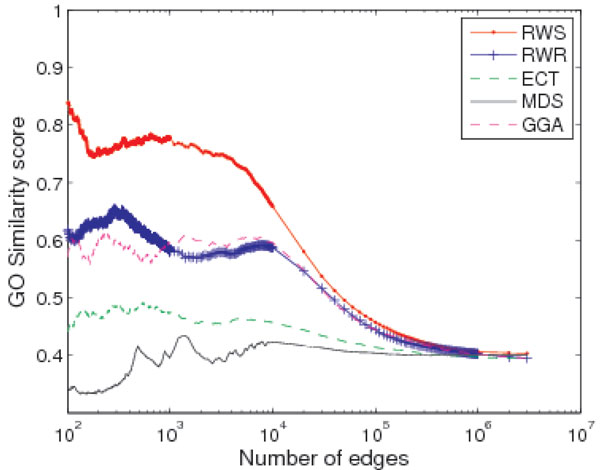
**Comparison with other algorithms on gene ontology based similarities**. Comparison between RWS and other network reconstruction algorithms. GO similarity score is the average GO value of the gene pairs with the top PP-TS scores.

We compared our algorithm with four existing methods, namely Euclidean commute time (ECT) [24], random walk with restart (RWR) [25], multiple dimensional scaling (MDS) [26], and global geometric affinity (GGA) [27]. The ECT and RWR methods are well-known in data mining and network analysis communities, while the MDS and GGA methods were recently proposed to improve the quality of PPI networks. All four algorithms calculate some topology-based similarity scores for pairs of nodes. Figure 2 shows the average GO similarity score of the top gene pairs in the PP-TS matrix. As shown in the figure, the average score generated by our method has the highest GO similarity.

**Figure 3:**
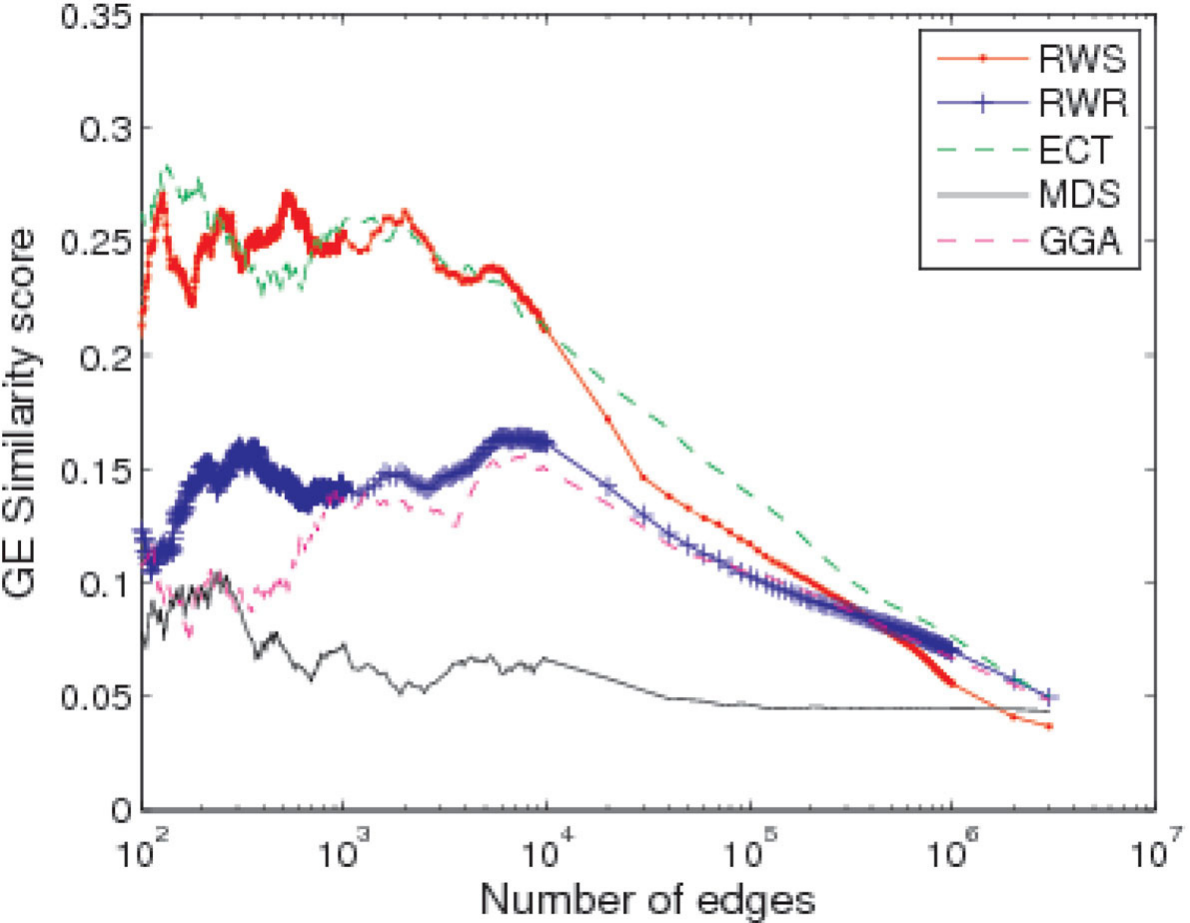
**Comparison with other algorithms on gene co-expression**. Comparison between RWS and other network reconstruction algorithms. GE similarity score is the average GE value of the gene pairs with the top PP-TS scores.

The average GE similarity scores are shown in Figure 3. Among all the 5 algorithms, both RWS and ECT have the best performance in gene co-expression score, however ECT has much lower GO similarity score compared to RWS as shown in Figure 2.

### Protein complex accuracy

**Figure 4:**
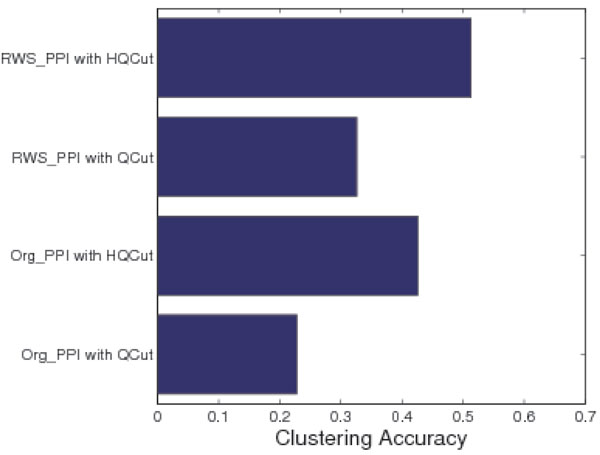
**Clustering Accuracy for Qcut algorithm and HQcut**. The figure demonstrates the clustering accuracy when Qcut and HQcut was applied to the original PPI network and RWS reconstructed PPI network.

As the PP-TS matrix is a weighted and completely connected network, thresholds values are used to convert it to a sparse network. To this end, we chose the cutoff value such that the average GO value of the interacting pairs in the modified network is the same as that of the original PPI network. This resulted in 26170 edges, as mentioned above. We then applied two network clustering algorithms, Qcut and HQcut, to the original and RWS modified networks, and compared the predicted complexes with the MIPS known protein complexes (see Methods). As shown in Figure 4, the prediction accuracy is significantly improved for both Qcut and HQcut on the RWS modified network compared to the original network, demonstrating that the network quality improvement by RWS is general. On the other hand, HQcut always achieves better accuracy than Qcut, due to HQcut's strategy in addressing the resolution limit problem.

**Figure 5:**
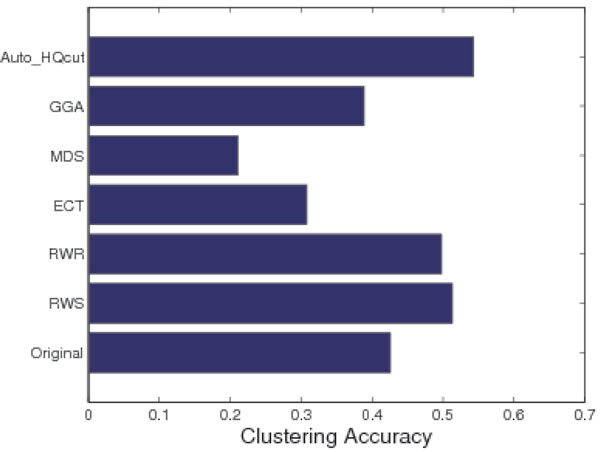
**HQcut clustering accuracy comparison**. The figure displays a comparison of clustering accuracy when applying HQcut to the original, RWS reconstructed, and other four algorithms reconstructed networks. These results are compared to the clustering accuracy when applying Auto-HQcut to the reconstructed RWS network.

Again, we compared our algorithm with the four existing methods, ECT, RWR, MDS and GGA. For a fair comparison, we choose a different cutoff for each algorithm so that the predicted PPI networks from different algorithms all have the same number of edges as the RWS modified PPI network. These networks are then subject to protein complex prediction using the HQcut algorithm. As shown in Figure 5, only RWS and RWR increased the complex prediction accuracy, and RWS is slightly better than RWR.

**Figure 6:**
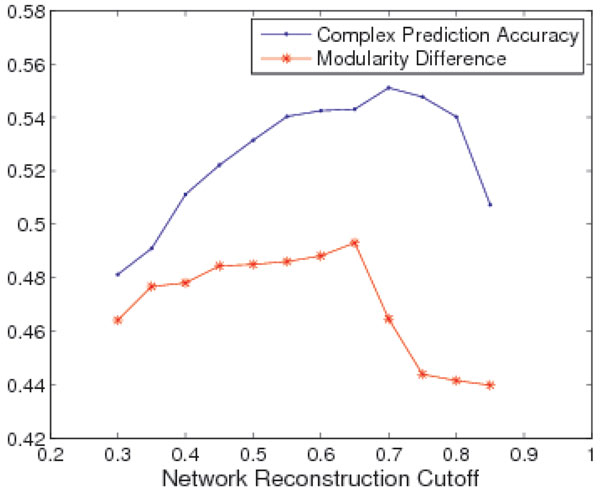
**Relationship between modularity difference value and complex prediction accuracy**. The modularity difference value and complex prediction accuracy show the same trend with different cutoffs.

In order to further improve the protein complex prediction accuracy of HQcut, we reconstructed a series of weighted PPI networks with different cutoffs, and run HQcut on each of the network to identify potential protein complexes. We also measured the modularity differences between the selected networks and random generated networks. As shown in Figure 6, the modularity difference is well correlated with the protein complex prediction accuracy. Using the automatically determined PP-TS similarity cutoff (0.65) at the largest modularity difference value (0.49), the corresponding complex prediction accuracy is close to the optimal prediction accuracy. As shown in Figure 6, RWS modified PP-TS matrix with the fully automated parameter determination HQcut (called Auto-HQcut) has significantly improved the prediction accuracy compared to other algorithms, demonstrating that both the RWS and Auto-HQcut can help improve complex prediction accuracy.

**Figure 7:**
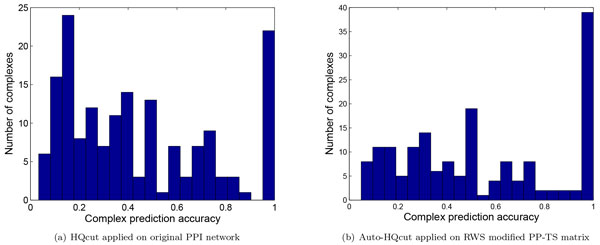
**Complex prediction accuracy distribution for the predicted complexes**.

Figure 7(a) and 7(b) show the accuracy distribution for the original PPI network and the RWS modified network with automatic parameter determination HQcut. Among the 170 known complexes, 77 clusters had increased accuracy, while 65 of them kept the same accuracy, and 28 clusters had decreased accuracy. As shown in Figure 7, the number of protein complexes with near perfect prediction is much improved from 22 complexes to 39 complexes. Also, there is a dramatic drop in the number of complexes at the accuracy level of 0.2 which is attributed to the more accurate prediction of the small size clusters. We also observed that most of the improved complexes are small in size.

**Figure 8:**
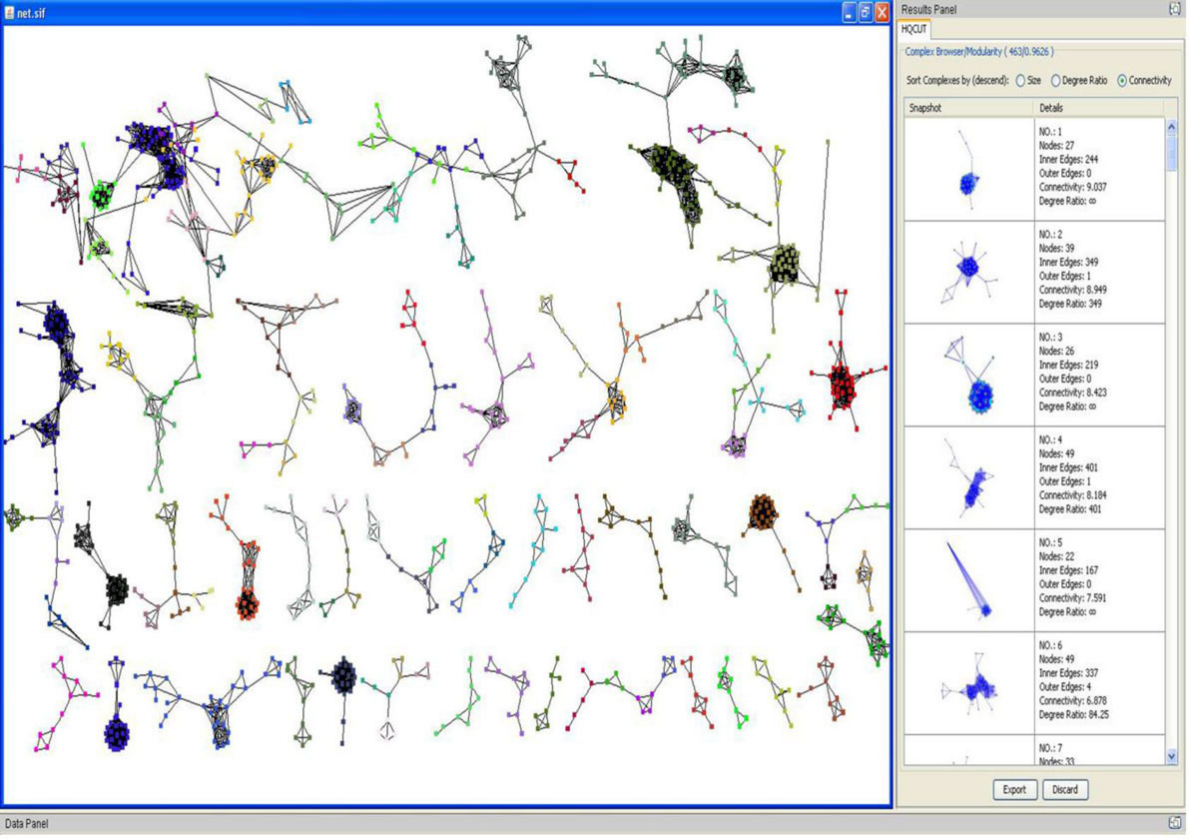
**Protein complexes identified with Auto-HQcut**. In the figure, the left panel displays the obtained network using a Cytoscpace plugin for HQcut and the right panel displays statistics about the network and the identified protein complexes.

To display the result of protein complexes obtained, we developed an interface to show the complexes and their associated properties. The result on Figure 8 shows some of the protein complexes obtained from using our approach. It can be seen the proteins belonging to the same complex are colored the same and are strongly connected. Protein belonging to different complexes are colored differently and the edges between complexes (outer edges) are fewer compared to edges within the complex (inner edges). More statistics related to the network and complexes obtained are displayed in the result panel for further exploration.

## Methods

### Protein-protein topological similarity matrix

Since the original PPI network is binary, incomplete in nature, and is not good for the clustering algorithms, we used the *Random Walk with Resistance*(RWS) [22] to reconstruct the network for better clustering performance.

Let *G*(*V*, *E*) be an undirected graph, *V *the set of vertices and *E *the set of edges. With the basic random walk idea, the probability to move from an initial node *v *to its neighbor node *u *in the next step is *Pvu *= 1*/d*(*v*) for (*v, u*) ∈ *E*, where *d*(*v*) is the degree of node *v*. In RWS algorithm, we introduced two types of resistance *ε *and *β *and the probability for a random walker initiated from node *v *to take the edge (*i, j*) at time point *k *+ 1 can be calculated by Equation (1), where

(1) $$f_{ij}^{\left(k+1\right)}=\{\begin{array}{ll} \max(0,q_{i}^{\left(k\right)}P_{ij}-\varepsilon ), & \text{if }q_{j}^{\left(k\right)}>0;\\ \max(0,q_{i}^{\left(k\right)}P_{ij}-\varepsilon ), & \text{if }q_{j}^{\left(k\right)}>0\text{ and}\;\max_{i}(q_{i}^{\left(k\right)}P_{ij})\geq \beta ;\\ 0,\text{ otherwise.} & \end{array}$$

and the probability for the random walker to reach node *j *at time point *k *+ 1 can be calculated as

(2) $$q_{{}^{j}}^{\left(k+1\right)}=\underset{i}{\sum }f_{ij}^{\left(k+1\right)}.$$

The RWS algorithm is a modified Random Walk algorithm which uses two types of resistances. The first type of resistance(*ε*) makes the final convergence status different for each node based on its topology within the network. The second type of resistance(*β*) is used to control the depth of a random walk and helps to avoid the hub node effect. When applying the RWS algorithm to the original PPI network, we obtain all the topological profiles for each node. These topological profiles are used to calculate the correlation between all pairs of nodes, where correlation value represents the topological similarity between two nodes. The combined topological similarity for all pairs of nodes builds the PP-TS matrix, which can be viewed as a network with weighted edges.

### Clustering procedure

In clustering the network, we first apply Qcut algorithm [19] which optimizes the modularity function (*Q*) of a given network [20]. However, because Qcut faces a resolution limit problem where communities smaller than a certain scale will never be identified, we further applied HQcut algorithm to the network to solve this problem. Even though HQcut works well, unlike the initial Qcut algorithm, a user has to input parameters to obtain the best Q value. Using HQcut, we devised Auto-HQcut which automatically picks the cutoff that gives the maximum Q value. A brief description of each algorithm is provided below.

#### Qcut algorithm

Qcut algorithm involves two steps: partitioning and refining. In partitioning, the network is recursively divided each time calculating the Q value until no further improvement is found in the Q value. In refining stage, the communities obtained in the partitioning stage are further refined to try to improve the Q value. In order to achieve this, the communities obtained are changed by: either moving the vertex from one community to another, combining two communities to form a new bigger community, or splitting a community to form two small communities. If the change in the original community results into a better Q value, a change is considered, otherwise no change is made to the original community.

#### HQcut algorithm

HQcut algorithm [19] improves Qcut algorithm by solving the resolution limit problem. It uses the Qcut algorithm to obtain the community structure which results in the highest Q value. To determine if the individual communities should further be partitioned, Qcut is applied again to the individual communities using threshold value that measures the strength of the community structure and the statistical significance of the original communities obtained. HQcut uses Q ≤ 0.3 threshold and p-value of 0.05 as a measure of community structure strength and its statistical significance respectively to decide whether the individual communities should further be partitioned and find whether partitioning would result in a better Q value.

#### Auto-HQcut

As seen from the algorithm description above, HQcut can further optimize the Q value hence increase prediction accuracy of protein complexes. However, it is hard to choose the best cutoff to apply on the PP-TS matrix that would produce the network with the best prediction accuracy of the protein complexes. As a result, we run HQcut to the networks generated from PP-TS matrix with different cutoffs, and measure the statistical significance of the resulting Q value by comparing the selected networks with the random generated networks. By using the modularity difference between these two values, we can tell if the selected network can predict the protein complexes from the random one. At the end, we pick the cutoff which gives the largest modularity difference value to be used to generate the protein complexes.

### Evaluations

#### Biological functional relevance

To evaluate the biological relevance of the newly predicted edges, we resort to gene ontology and gene expression, which are widely used in the functional evaluation. To measure the biological functional relevance between any pair of genes, we used the semantic similarity between the GO terms annotated with the proteins, with a popular method [28,29], which incorporates both a global metric and a local metric for balance and consistency. Results shown in this paper are based on the "Molecular Function" branch of Gene Ontology. Using "Biological Process" yielded very similar values, and "Cellular Localization" resulted in slightly lower but consistent values. We also measured the Pearson correlation coefficient between the gene expression profiles of every pair of genes using the yeast stress response microarray data [30]. With the results from above two methods, we can use the GO/GE similarity between a pair of nodes to represent the biological relevance of those two proteins.

#### Complex prediction accuracy

To investigate the protein complexes prediction accuracy improvement, we compared the predicted complexes with the MIPS known protein complexes [31], which include 767 proteins in 170 known complexes after intersecting with the PPI network. To measure the accuracy of the prediction, we used the Fowlkes-Mallows index for comparing clustering [32,33]. Formally, let *A *be the list of gene pairs that fall into the same complex in the set of predicted complexes and *B *be the list of gene pairs in the set of known complexes, the prediction accuracy is measured by $|A\cap B|/\sqrt{\left|A|\times |B\right|}$, where *|A| *denotes the cardinality of the set *A*.

### Visualization

To help visualize protein complexes obtained from our approach, we developed an interface also used as a plugin in Cytoscape [34]. The interface was developed in MATLAB and compiled into java classes using MATLAB java compiler. The interface displays the interaction of the obtained protein complexes and the properties of the protein complexes such as average connectivity, ratio between intra-cluster edges and intercluster edges, and the number of proteins within the complex. One unique feature of the interface is its ability to uniquely color the protein complexes so that different complexes within the network can easily be differentiated.

## Conclusion

In this paper, we developed a random walk based algorithm that converts the PPI network into a protein-protein topological similarity matrix which is then used to construct weighted networks. The key idea is that two proteins sharing some high-order topological similarities are likely to be interacting with each other and be involved in the same biological processes. Using the reconstructed weighted network, we can measure the interactions of all the protein-protein pairs using a real value as opposed to the "connected/non-connected" measure in the original PPI network, which also helps to reduce noise and uncover significant biological interactions that would otherwise be overlooked when analyzing the original PPI network. We then used a parameter free modularity based community finding algorithm (Auto-HQcut) to identify protein complexes from PPI network by optimizing the modularity function. Finally, we used the interface we developed to visualize and analyze the characteristics of the protein complexes obtained. The results showed that the algorithm can find higher modularity protein complexes with better prediction accuracy.

In summary, the weighted network reconstructed from PP-TS matrix has much higher biological relevance than the original network and Auto-HQcut significantly improved protein complex prediction accuracy. Since our method improved the protein-protein similarity quality without any additional biological information involved, the algorithm can be easily combined with other approaches to improve the analysis of PPI networks and protein complex prediction.

## Competing interests

The authors declare that they have no competing interests.

## Authors' contributions

CL and JR conceived the research. CL implemented the RWS algorithm and performed the experiments. ST designed and implemented the visualization interface. AJRB provided biological background. CL, JR, ST and AJRB analyzed the data. CL, ST and JR wrote the manuscript. All authors read and approved the final manuscript.

## Declarations

The publication costs for this article were funded by National Institutes of Health (grant number SC3GM086305).

This article has been published as part of *Proteome Science *Volume 11 Supplement 1, 2013: Selected articles from the IEEE International Conference on Bioinformatics and Biomedicine 2012: Proteome Science. The full contents of the supplement are available online at http://www.proteomesci.com/supplements/11/S1.

## References

[B1] Przulj N Protein-protein interactions: making sense of networks via graph-theoretic modeling BioEssays 2011 11 2 115 123 10.1002/bies.201000044 21188720

[B2] Bader G Hogue C Analyzing yeast protein-protein interaction data obtained from different sources Nat Biotechnol 2002 11 991 7 10.1038/nbt1002-991 12355115

[B3] Wang C Ding C Yang Q Holbrook S Consistent dissection of the protein interaction network by combining global and local metrics Genome Biol 2007 11 R271 10.1186/gb-2007-8-12-r271 18154653 PMC2246273

[B4] King A Przulj N Jurisica I Protein complex prediction via cost-based clustering Bioinformatics 2004 11 3013 20 10.1093/bioinformatics/bth351 15180928

[B5] Asthana S King O Gibbons F Roth F Predicting protein complex membership using probabilistic network reliability Genome Res 2004 11 1170 1175 10.1101/gr.2203804 15140827 PMC419795

[B6] Wang J Li M Deng Y Pan Y Recent advances in clustering methods for protein interaction networks BMC Genomics 2010 11 Suppl 3 S10 10.1186/1471-2164-11-S3-S10 21143777 PMC2999340

[B7] Ulitsky I Shamir R Identifying functional modules using expression profiles and confidence-scored protein interactions Bioinformatics 2009 11 1158 64 10.1093/bioinformatics/btp118 19297352

[B8] Chua HN Sung WK Wong L Exploiting indirect neighbours and topological weight to predict protein function from protein-protein interactions Bioinformatics 2006 11 13 1623 1630 http://www.hubmed.org/display.cgi?uids=16632496 10.1093/bioinformatics/btl145 16632496

[B9] Sharan R Ulitsky I Shamir R Network-based prediction of protein function Molecular Systems Biology 2007 11 88 17353930 10.1038/msb4100129 PMC1847944

[B10] Friedel C Krumsiek J Zimmer R Bootstrapping the Interactome: Unsupervised Identification of Protein Complexes in Yeast Journal of Computational Biology 2009 11 8 1 17 19630542 10.1089/cmb.2009.0023

[B11] Lee K Chuang H Beyer A Sung M Huh W Lee B Ideker T Protein networks markedly improve prediction of subcellular localization in multiple eukaryotic species Nucleic Acids Res 2008 11 e136 10.1093/nar/gkn619 18836191 PMC2582614

[B12] Chuang HY Lee E Liu YT Lee D Ideker T Network-based classification of breast cancer metastasis Mol Syst Biol 2007 11 140 140 http://www.hubmed.org/display.cgi?uids=17940530 17940530 10.1038/msb4100180 PMC2063581

[B13] Hannum G Srivas R Guenole A van Attikum H Krogan N Karp R Ideker T Genome-wide association data reveal a global map of genetic interactions among protein complexes PLoS Genet 2009 11 e1000782 10.1371/journal.pgen.1000782 20041197 PMC2788232

[B14] Ideker T Sharan R Protein networks in disease Genome Res 2008 11 644 52 10.1101/gr.071852.107 18381899 PMC3863981

[B15] Kim Y Wuchty S Przytycka T Identifying causal genes and dysregulated pathways in complex diseases PLoS Comput Biol 2011 11 e1001095 10.1371/journal.pcbi.1001095 21390271 PMC3048384

[B16] Hidalgo C Blumm N Barabasi A Christakis N A dynamic network approach for the study of human phenotypes PLoS Comput Biol 2009 11 e1000353 10.1371/journal.pcbi.1000353 19360091 PMC2661364

[B17] Huang H Jedynak BM Bader JS Where Have All the Interactions Gone? Estimating the Coverage of Two-Hybrid Protein Interaction Maps PLoS Comput Biol 2007 11 11 e214 10.1371/journal.pcbi.0030214 18039026 PMC2082503

[B18] Lei C Ruan J A novel link prediction algorithm for reconstructing protein-protein interaction networks by topological similarity Bioinformatics 2013 11 3 355 364 10.1093/bioinformatics/bts688 23235927 PMC3562060

[B19] Ruan J Zhang W Identifying network communities with a high resolution Physical Review E 2008 11 016104 18351912 10.1103/PhysRevE.77.016104

[B20] Newman M Modularity and community structure in networks Proc Natl Acad Sci USA 2006 11 8577 82 10.1073/pnas.0601602103 16723398 PMC1482622

[B21] Fortunato S Barthelemy M Resolution limit in community detection Proc Natl Acad Sci USA 2007 11 36 41 10.1073/pnas.0605965104 17190818 PMC1765466

[B22] Lei C Ruan J A random walk based approach for improving protein-protein interaction network and protein complex prediction Bioinformatics and Biomedicine (BIBM), IEEE International Conference on 2012 2012 1 6

[B23] Krogan N Cagney G Yu H Zhong G Guo X Ignatchenko A Li J Pu S Datta N Tikuisis AP Punna T Peregrn-Alvarez J Sha les M Zhang X Davey M Robinson M Paccana ro A Bray J Sheung A Beattie B Richards D Canadien V Lalev A Mena F Wong P Starostine A Canete M Vlasblom J Wu S Or si C Collins S Chandran S Haw R Rilstone J Gandi K Thompson N Musso G St Onge P Ghanny S Lam M Butland G Altaf-Ul AM Kanaya S Shilatifard A O'Shea E Weissman JS Ingles C Hughes T Parkinson J Gerstein M Wodak S Emili A Greenblatt J Global landscape of protein complexes in the yeast Saccharomyces cerevisiae Nature 2006 11 637 643 10.1038/nature04670 16554755

[B24] Fouss F Pirotte A Renders JM Saerens M Random-Walk Computation of Similarities between Nodes of a Graph with Application to Collaborative Recommendation IEEE Transactions on Knowledge and Data Engineering 2007 11 3 355 369

[B25] Tong H Faloutsos C Pan JY Fast Random Walk with Restart and Its Applications Proceedings of the Sixth International Conference on Data Mining, ICDM '06 2006 Washington, DC, USA 613 622 IEEE Computer Society

[B26] Kuchaiev O Rasajski M Higham DJ Przulj N Geometric De-noising of Protein-Protein Interaction Networks PLoS Comput Biol 2009 11 e1000454 10.1371/journal.pcbi.1000454 19662157 PMC2711306

[B27] Fang Y Benjamin W Sun M Ramani K Global Geometric Affinity for Revealing High Fidelity Protein Interaction Network PLoS ONE 2011 11 5 e19349 10.1371/journal.pone.0019349 21559288 PMC3086913

[B28] Wang J Du Z Payattakool R Yu P Chen C A new method to measure the semantic similarity of GO terms Bioinformatics 2007 11 1274 81 10.1093/bioinformatics/btm087 17344234

[B29] Yu G Li F Qin Y Bo X Wu Y Wang S GOSemSim: an R package for measuring semantic similarity among GO terms and gene products Bioinformatics 2010 11 976 8 10.1093/bioinformatics/btq064 20179076

[B30] Gasch A Spellman P Kao C Carmel-Harel O Eisen M Storz G Botstein D Brown P Genomic expression programs in the response of yeast cells to environmental changes Mol Biol Cell 2000 11 4241 4257 10.1091/mbc.11.12.4241 11102521 PMC15070

[B31] Mewes H Frishman D Mayer K Munsterkotter M Noubibou O Pagel P Rattei T Oesterheld M Ruepp A Stumpflen V MIPS: analysis and annotation of proteins from whole genomes in 2005 Nucleic Acids Res 2006 11 D169 172 10.1093/nar/gkj148 16381839 PMC1347510

[B32] Meila M Comparing clusterings: an axiomatic view ICML '05: Proceedings of the 22nd international conference on Machine learning 2005 New York, NY, USA: ACM Press 577 584

[B33] Fowlkes E Mallows C A method for comparing two hierarchical clusterings J Amer Statist Assoc 1983 11 553 569 10.1080/01621459.1983.10478008

[B34] Shannon P Markiel A Ozier O Baliga NS Wang JT Ramage D Amin N Schwikowski B Ideker T Cytoscape: a software environment for integrated models of biomolecular interaction networks Genome research 2003 11 11 2498 2504 10.1101/gr.1239303 14597658 PMC403769

